# Greed communication predicts the approval and reach of US senators’ tweets

**DOI:** 10.1073/pnas.2218680120

**Published:** 2023-03-06

**Authors:** Eric J. Mercadante, Jessica L. Tracy, Friedrich M. Götz

**Affiliations:** ^a^Department of Psychology, University of British Columbia, Vancouver, BC V6T 1Z4, Canada

**Keywords:** greed, social media, political psychology, psycholinguistics, Twitter

## Abstract

Social media are at the forefront of modern political campaigning. They allow politicians to communicate directly with constituents and constituents to endorse politicians’ messages and share them with their networks. Analyzing every tweet of all US senators holding office from 2013 to 2021 (861,104 tweets from 140 senators), we identify a psycholinguistic factor, greed communication, that robustly predicts increased approval (favorites) and reach (retweets). These effects persist when tested against diverse established psycholinguistic predictors of political content dissemination on social media and various other psycholinguistic variables. We further find that greed communication in the tweets of Democratic senators is associated with greater approval and retweeting compared to greed communication in the tweets of Republican senators, especially when those tweets also mention political outgroups.

With increasing political polarization, the course of American politics can shift dramatically depending on who holds power (1). Politicians therefore need every vote they can muster to attain and hold majorities, especially since thin margins can decide elections; for example, one senate race in the 2022 midterm elections (senate seat for Nevada) was decided by only 0.5% (2). In this ultracompetitive environment, social media platforms like Twitter have become a valued avenue for politicians to reach their constituents and for constituents to spread politicians’ messages (3). Politicians, therefore, have a strong incentive to post content that their existing base not only appreciates but shares with their own followers, who might then start following and supporting the politician themselves.

Certain language patterns lead social media users to amplify—that is publicly endorse (“favorite”) and share (“retweet”)—political content. For example, tweets mentioning political outgroups (3) and employing moral–emotional (4) or negative emotion language (5) are more likely to be shared. Here, we examined whether this phenomenon extends to language about a psychological and emotional construct seen as central to policy- and decision-making: greed.

Psychologically, greed is “the desire to acquire more and the dissatisfaction of never having enough” (6, p. 519). Several influential philosophers, including Marx, Hobbes, and Machiavelli, argue that coping with the ubiquitous human tendency toward greed is an inevitable challenge of social living and—in turn—that political leaders and institutions need to curtail individual greed for the betterment of society (7). In contrast, others—such as Adam Smith—view greed as a productive and ultimately functional force in society because the motivation to amass personal wealth drives greedy people to invent new products and solve important problems that benefit society as a whole (7). Nonetheless, greed also predicts engaging in unethical behaviors to acquire resources (8, 9) and is often implicated in poor financial outcomes. For instance, chief executive officers' greed predicts lower shareholder returns (10) and slower recovery of stock prices after the 2008 financial crisis (9). Consequently, greed may be viewed negatively by the public, despite its potential for positive outcomes in a capitalist society. Politicians who explicitly discuss the detrimental consequences of greed might therefore be advantaged. Accordingly, we hypothesized that US Senators’ tweets discussing greed would receive more favorites and retweets (hypothesis 1), which might increase these senators’ followership among the electorate (11).

## Results

**Fig. 1.**
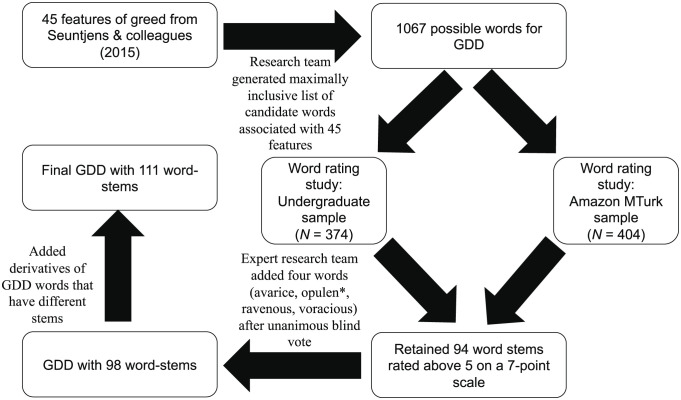
Preregistered GDD development procedure. Notes: For full details on the GDD development procedure, see *SI Appendix*. * indicates that any letters following the stem are counted in the GDD (e.g., “opulen*” covers opulent and opulence).

We developed and validated a novel greed descriptive dictionary (GDD) (Fig. 1, *Materials and Methods*, and *SI Appendix* for full details) and used it to examine associations between greed language and the number of favorites and retweets received on all tweets from all sitting US Senators between January 3, 2013, and December 28, 2021 (*N* = 861,104 tweets from 140 Senators). We examined greed language within individual tweets (henceforth “tweet level”) and each senator’s average level of greed language (“senator level”). Effects at the tweet level were senator-mean-centered and thus reflect the impact of greed language for any given tweet, normed to the tweeter (i.e., does a tweet with more greed language receive more amplification and approval than a tweet with less greed language *from the same senator?*). In contrast, effects at the senator level reflect the impact of senators’ general tendency to tweet about greed (i.e., does any given tweet from a senator who tends to tweet about greed receive more amplification and approval than a tweet from a senator who tweets less about greed?).

The use of greed language predicted more favorites and retweets, at both the tweet level (*β_Favorites _* = 0.021, *P * < 0.001; *β_Retweets _*= 0.035, *P * < 0.001) and senator level (*β_Favorites _* = 0.30, *P *< 0.001; *β_Retweets _*= 0.25, *P *< 0.001; Fig. 1). Effect sizes were comparable to those of other linguistic variables predicting political engagement at the tweet level (3–5). We examined the relationships between greed language and emotion language (12) to test whether senators are more likely to describe greed as something positive or negative. At both the tweet and senator levels, follow-up analyses showed that greed language co-occurred with lesser positive emotional language (*r_tweet-level _*= −0.062*, P *< 0.001; *r_senator-level _*= −0.39, *P *< 0.001), greater negative emotional language (*r_tweet-level _*= 0.011, *P *< 0.001; *r_senator-level _*= 0.40, *P *< 0.001), and a more negative overall emotional tone (*r_tweet-level _*= −0.042, *P *< 0.001; *r_senator-level _*= −0.43, *P *< 0.001), and these results were consistent across parties.^*^ These analyses suggest that senators on both sides of the aisle tend to portray greed as a destructive force in society when they tweet about it.

**Fig. 2.**
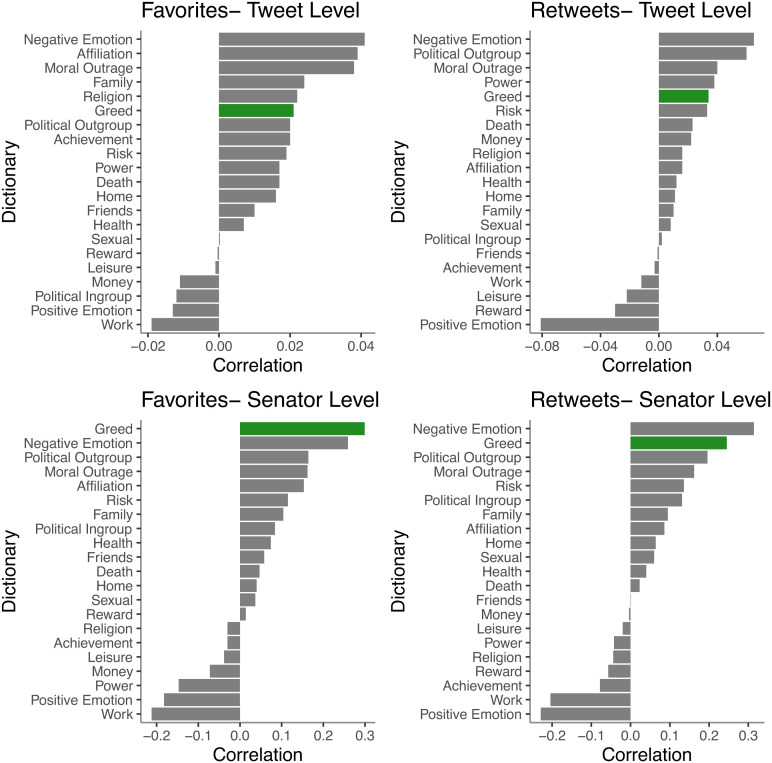
Correlations between each linguistic dictionary examined and favorites (*Left* column) and retweets (*Right* column), at tweet level (*Top* row) and senator level (*Bottom* row). Notes: At the tweet level, effect sizes greater than *r* = ±0.001 are significant, *P* < 0.05. At the senator level, effect sizes greater than *r* = ±0.085 are significant, *P* < 0.05.

To contextualize the observed effects and test their robustness, we examined several other linguistic variables that a) capture politically salient topics (e.g., health, family, religion), b) have been shown to predict engagement with political tweets in past research [in-group and out-group language (3), moral–emotional language (4), positive and negative emotion language (5)], or c) capture concepts related to greed (e.g., money, work, and risk); see Fig. 2. Of the 21 variables examined, greed was the strongest predictor of senator-level favorites, the second strongest predictor of senator-level retweets, the sixth strongest predictor of tweet-level favorites, and the fifth strongest predictor of tweet-level retweets. In a model including all of these linguistic variables at both the tweet and senator levels, greed effects held for both outcomes at both levels (tweet level: *β_Favorites _*= 0.024, *P *< 0.001; *β_Retweets _*= 0.028, *P *< 0.001, senator level: *β_Favorites _*= 0.28, *P *< 0.001; *β_Retweets _*= 0.11, *P *= 0.03).

We next tested whether greed language is more impactful for Democrats or Republicans. We found significant interactions between greed language and the party of the senator tweeting for favorites and retweets at the tweet level (*β_Favorites _*= −0.025, *P *< 0.001; *β_Retweets _*= −0.016, *P *< 0.001) but not the senator level (*β_Favorites _*= −0.05, *P *= 0.62; *β_Retweets _*= −0.10, *P *= 0.26). Decomposing the tweet-level interactions revealed that greed language was a significantly stronger predictor of tweet-level favorites and retweets for Democratic senators (*β_Favorites _*= 0.031, *P *< 0.001; *β_Retweets _*= 0.041, *P *< 0.001) than Republican senators (*β_Favorites _*= 0.006, *P *< 0.001; *β_Retweets _*= 0.025, *P *< 0.001).

Finally, since greed is socially undesirable and outgroup animosity is known to predict retweets (3), greed language might be provocative because senators use it to derogate their political opponents. For example, the following tweet from Senator Van Hollen (Democrat-Maryland) is high in both greed and outgroup language (+2 SD on both; 6.7% greed language, 4.4% outgroup language) and was one of the most retweeted tweets in the dataset (+5 SD; retweeted 16,107 times):

“Bad news. After saying they wanted to join us in helping workers, families, and small + midsized businesses that are going under, Trump and McConnell have taken a total u-turn. They just want to bail out big corporate cronies at everyone else’s expense. Unacceptable.” (Tweeted on March 22, 2020)

In fact, significant interactions emerged between greed and political outgroup language for tweet-level favorites and retweets for Democrats (*β_Favorites _*= 0.005, *P *< 0.001; *β_Retweets _*= 0.004, *P *< 0.001) but not Republicans (*β_Favorites _*= −0.001, *P *= 0.23; *β_Retweets _*= −0.002, *P *= 0.15). Tweets from Democrats mentioning greed (+1 SD) and political outgroups (+1 SD) were significantly more likely to be favorited (*β_Favorites _*= 0.037, *P *< 0.001) and retweeted (*β_Retweets _*= 0.045, *P *< 0.001) (−1 SD; *β_Favorites _*= 0.028, *P *< 0.001; *β_Retweets _*= 0.037, *P *< 0.001).^†^

## Discussion

Using social media to captivate like-minded partisans and motivate them to act can decide elections (11). It also can play a role in elevating some politicians to stardom (e.g., Alexandria Ocasio-Cortez) while ushering others from the political arena [e.g., Joe Crowley, the 10-term incumbent unseated by Ocasio-Cortez; (13)]. Our research suggests that highlighting greed in tweets is associated with an increase in amplification and approval of political messages by US senators on social media and that this association a) occurs regardless of political, moral, and emotional framings (3–5), b) emerges across party lines, and c) is especially advantageous for Democrats when used to attack political opponents.

## Materials and Methods

The data, dictionary development protocol, code, and materials are available on our Open Science Framework (OSF) repository (*SI Appendix*): https://bit.ly/3fTheQd.

This research was approved by the Behavioral Research Ethics Board of University of British Columbia (Protocol #H21-00679). Participants in dictionary development studies provided informed consent after reading a consent form prior to participating.

To quantify greed language, we developed the GDD informed by the method of Lawson et al. [(14); preregistered at https://bit.ly/3fTheQd]. Fig. 1 displays a flow chart of this process. The complete GDD can be found at https://bit.ly/3CW2edo; additional methodological and validation details are in *SI Appendix*. To test for construct validity, we compared trained coders’ ratings of greed communication in a sample of 1,087 tweets that included the word “money” with GDD scores of these tweets. The GDD corresponded strongly with coders’ ratings (β = 0.49, *P *< 0.001). To test for convergent validity, we preregistered predictions of positive associations between the GDD and 13 theoretically relevant dictionaries from Linguistic Inquiry and Word Count-22 (LIWC-22; https://bit.ly/3rWOw3N) in a) the senators’ tweets used in the main analyses and b) 2,225 publicly available BBC articles (https://www.kaggle.com/c/learn-ai-bbc). Of our 13 predictions, 9 were supported in the BBC data, and 10 (including the 9 supported in the BBC data) were supported in the tweets.

We used previously developed dictionaries to assess political in-group and out-group language (3) and moral–emotional language (4). The remaining variables measured came from LIWC-22 (12).

Tweets were scraped using the AcademicTwitteR package in R (15). We scraped all tweets from January 3, 2013 [the first day of the 113th session of Congress and approximately the time when social media became a popular tool for politicians and all senators had Twitter accounts; (16)] to December 28, 2021 (approximately halfway through the 117th session of Congress and prior to a recess) from all US senators who held the office during any portion of that timeframe.

We first preprocessed the tweets following standard text analysis protocols [(3–5, 14); see https://bit.ly/3DCMZXq]. We conducted mixed linear effects models with tweets nested within senators. Consistent with past research, we applied a reciprocal transformation to both favorites and retweets to account for skewness (5). All variables were standardized.

## Supplementary Material

Appendix 01 (PDF)

## Data Availability

Twitter data, data from word rating studies, have been deposited in Open Science Framework (17) https://osf.io/7r2bc/?view_only=80b3424277734ba59f53fff506e5eb18.

## Footnotes

^*^Correlations between greed and emotion language are as follows: for Republicans, positive emotions (*r_tweet-level_ *= −0.070*, P *< 0.001; *r_senator-level_ *= −0.32, *P *< 0.001), negative emotions (*r_tweet-level _*= 0.004*, P *= 0.009; *r_senator-level _*= 0.34, *P *< 0.001), overall emotional tone (*r_tweet-level _*= −0.050*, P *< 0.001; *r_senator-level _*= −0.28, *P *< 0.001); for Democrats, positive emotions (*r_tweet-level _*= −0.056*, P *< 0.001; *r_senator-level _*= −0.43, *P *< 0.001), negative emotions (*r_tweet-level _*= 0.013*, P *< 0.001; *r_senator-level _*= 0.37, *P *< 0.001), overall emotional tone (*r_tweet-level _*= −0.034*, P *< 0.001; *r_senator-level _*= −0.53, *P *< 0.001).
