# Supplementary material for: Greed communication predicts the approval and reach of US senators’ tweets
Source: Proc Natl Acad Sci U S A. 2023 Mar 6;120(11):e2218680120. doi: 10.1073/pnas.2218680120 (PMC10089212; doi:10.1073/pnas.2218680120)
Supplement: Supplementary file 1 — Appendix 01 (PDF) [file pnas.2218680120.sapp.pdf]

Supporting Information for "Greed communication predicts the approval and reach of US senators' tweets"

Detailed Description of Dictionary Development Process

Before completing any of the following steps, we pre-registered the protocol for developing the greed descriptive dictionary (GDD; https://bit.ly/3rUwbEj). Our approach was inspired and informed by Lawson and colleagues' (1) procedure for developing agency and communion dictionaries.

Word Generation

We began with 45 features of greed identified by Seuntjens and colleagues (2). In their research, they used prototype analysis (3; see 4, 5, and 6 for examples) to develop a bottom-up definition of greed, which they ultimately formulated as "the desire to acquire more and the dissatisfaction with never having enough" (2, p. 519). In the process, they identified 45 central features (e.g., acquisitiveness, selfishness, materialism) and peripheral features (e.g., pride, thriftiness, sloth) of greed (see https://bit.ly/3yHuY79 for all 45 features). Based on these features, the lead author and the senior author of the current manuscript and a team of six research assistants each independently generated every related word and phrase to each feature that came to mind. After removing duplicates and consolidating various forms of the same word into one root word (e.g., "want", "wanted", "wants", and "wanting" were all reduced to "want"), this resulted in 1,067 candidate words and phrases (i.e., multiple words that count as one coherent entry in the GDD; e.g., "too much", "looking out for oneself") for the GDD.

Word Selection

To arrive at a final selection of words for the GDD, we conducted two studies in which participants rated each word and phrase for the extent to which it is related to greed. The study protocols were approved by the Behavioral Research Ethics Board at the University of British Columbia (Protocol #H21-00679). Both studies were conducted in Spring of 2021. One study used an undergraduate sample (N = 409), and one study used a sample of American adults recruited through Amazon Mechanical Turk (N = 404). In both studies, each participant rated 175 randomly selected words from the full list of 1,067 candidate words on their relation to greed. Participants used a rating scale ranging from 1 (Not at all related to greed) to 7 (Extremely related to greed). We chose this sample size and this number of words per participant so that each word would be ultimately rated by at least 50 individuals.

As pre-registered, we planned to include the 100 top-rated words in the final GDD, but we also stipulated that each word must have an average rating of at least 5 to be included. We aggregated the data from Studies 1 and 2 (see https://bit.ly/3g63T7d for the ratings from each study separately and aggregate data). With the aggregate data, 114 words were rated above 5. We further reduced this list by removing words with common stems (e.g., "egoism" and "egomaniac" were consolidated into "ego*"; * denotes that anything with that stem is counted as a GDD word). In addition, preliminary analyses showed that text sources that included lists of prices were rated as highly greedy because of the inclusion of "$" in the GDD, so we removed this symbol (although "$" was rated as highly related to greed, it carries a notably different meaning when evaluated in isolation, as was done in these studies, compared to how it is typically used in texts-- i.e., to denote a dollar value). This resulted in a final list of 94 unique stems that were rated above 5 in the aggregate data.

As pre-registered, in a mutually blind expert committee, the three authors next each individually reviewed the final list and identified additional words that did not make the final list but that they thought were integral to the construct of greed. As pre-registered, any words added to the GDD had to be independently generated by all three authors (https://bit.ly/3rUwbEj). We chose to incorporate this step into our dictionary development process because expert opinion helps ensure that the finalized dictionary corresponds to expert conceptualizations of the construct of interest. In fact, this is an approach that is commonly used in dictionary development projects (e.g., 1, 7, 8). For example, expert opinion was a crucial feature in the development of dozens of dictionaries included in the leading software for language analysis in the field, LIWC-22 (7). In building these dictionaries, expert judges indicated whether each of the candidate words for each construct of interest should be included, and words were ultimately included only if a majority of these judges agreed. We therefore planned –as described in our pre-registration document— to "complement [the] data-driven approach" by "[adding] words to the dictionary based on theoretical conceptions of greed... that were not previously identified" (see https://bit.ly/3H4z7Hv, p. 1). However, to avoid adding words generated on the basis of individual authors' idiosyncratic beliefs, we pre-registered our plan to add new words only if all three authors independently agreed on them.

This resulted in the addition of four words to the GDD— avarice, opulence (and all derivatives (e.g., opulent)), ravenous, and voracious; all three authors independently judged each of these words as directly relevant to greed, consistent with their dictionary definitions (dictionary.com):

- Avarice: insatiable greed for riches; inordinate, miserly desire to gain and hoard wealth.
- Opulence: wealth, riches, or affluence. Abundance, as of resources or goods; plenty
- Ravenous: extremely hungry; famished; voracious; extremely rapacious; intensely eager for gratification or satisfaction.
- Voracious: craving or consuming large quantities of food; exceedingly eager or avid.

Despite their relevance to greed, each of these words received a greed-relevance rating below the cut-off point (5 out of 7 on a Likert scale) in our studies, presumably because these four words are infrequently used in language today. To verify this assumption, we used Google Ngram to examine the frequency of these four words in Google's Google Books database, which includes over 200 billion words from books written in English between 1800 and 2019. For the sake of comparison, we also examined the frequencies of the four words/phrases that were rated highest on greed relevance (i.e., greed, money-hungry, never enough, never satisfied; for word pairs such as "money-hungry" we examined the frequency of both components (e.g., "money" and "hungry") because participants who understand these words in isolation would understand the full phrase).

According to Google Ngram, the four added words are very uncommon in recent books written in English, compared with the highly rated words. More specifically, in 2019, "avarice" comprised .000092% of the words in Google Books' corpus for that year (i.e., books written in English published in 2019), "opulent" comprised .000093%, "ravenous" comprised .000090%, and "voracious" comprised .000066%. In contrast, the six words comprising the four highest rated words/phrases in these studies all have frequencies of .00043% or greater in 2019 (greed =

.00043%, money = .017%, hungry = .0020%, never = .044%, enough = .025%, satisfied = .0026%), which is 4.67 times more frequent than the most frequently used of the added words (opulent); this difference supports our assumption that the added words were not rated above the greed-relevance cut-off because they were less familiar to participants. Nonetheless, some portion of participants did understand these words, because each was rated only slightly below the cut-off point across both studies, and each was rated greater than one standard deviation above the mean of all words rated (M = 3.63, SD = 1.01): avarice = 4.90, opulence = 4.82, ravenous = 4.81, voracious = 4.78.

It is also noteworthy that, despite their rarity in current common language, including the additional four words in the dictionary ensures that the GDD will be applicable to a broad range of data sources, including historical texts. Indeed, the Google Ngram analyses show that "avarice" and "opulent" were used much more frequently in 1800 (.0013% and .00081% of words in books published in 1800, respectively) than today (.000092% and .000093%, respectively). In other words, "avarice" was used 14.13 times as frequently in 1800 than in 2019, and "opulent" was used 8.71 times more frequently in 1800 than in 2019. It was partly with this goal in mind that we planned to use expert additions as needed.

Finally, we also expanded the dictionary with forms of included words that do not share the same stem (e.g., we added "stole" as a separate word from "steal" to account for references to this word in the past tense). The final GDD thus contains 111 word stems (e.g., "manipulat*" is used to capture "manipulate", "manipulated", "manipulating", etc.), and can be found here: https://bit.ly/3CW2edo.

Dictionary Validation

Construct Validity

To assess the construct validity of the GDD when used on tweets, four advanced undergraduate research assistants were trained on the psychological definition of greed [i.e., "the desire to acquire more and the dissatisfaction with never having enough" (2; p. 519)]. We then scraped 1,200 tweets that included the keyword "money" from all public twitter accounts, and we specified that the tweets must have been tweeted between January 1, 2021 and March 16, 2021. We chose these dates with the goal of scraping approximately 1,000 usable tweets (i.e., some might not be written completely in English), and after removing unusable tweets, we were left with 1,087 tweets for coding. This is a manageable number for coders to read and rate for greed (each coder rated half of the tweets; 543-544 tweets), and that would also provide a large enough sample size for highly powered analyses (1,087 tweets provided 95% power to detect an effect of size r = .125 in multiple regression (9); see below). We aimed to scrape tweets posted within a short timeframe (3 months) to maximize similarity in temporal context and linguistic conventions (e.g., the kinds of slang terms that are used) among tweets. Given that we scraped tweets from all public Twitter accounts, all 1,087 were posted within the same two days prior to scraping (March 15, 2021- March 16, 2021).

We targeted tweets that included the word "money" to obtain a sample of tweets that varied substantially in the extent to which they discussed greed. We stipulated that none of the scraped tweets were retweets of another user, replies to another user, or quote tweets of another user, and also that the tweets did not include mentions of another user or any media (e.g., photos, videos, etc.). Undergraduate coders then read each tweet and rated the extent to which it discussed greed from 1 (Not at all) to 5 (Extremely). An example of a tweet that was rated as a 1 was "Lemon and avocado grower saves money in water efficiency, pesticides and fertilizer" and a tweet rated as a 5 was "All I want is the money, I don't want anything else".

Consistent with past research and the leading software for text analysis in the field (LIWC; 7), GDD scores were calculated as the percentage of words in each tweet that were in the GDD.

To determine construct validity, we measured the correspondence between coders' ratings of each tweet and GDD scores of those tweets. Given that GDD scores represent the percentage of GDD words out of the total number of words used and tweets tend to be short (the average tweet in this dataset was 21 words), GDD scores are highly affected by the total number of words in the tweet. We therefore included an interaction term with tweet length to test whether the association between the dictionary and coders' ratings depends on tweet length. This analysis revealed both a main effect of the GDD (β=.49,p<.001), indicating strong correspondence between the GDD and coders' ratings of greed for a tweet of average length, and an interaction with tweet length (β=.32,p<.001), indicating that GDD scores more strongly corresponded to coders' ratings for longer tweets (+1 SD; β=.81,p<.001), and less strongly but still significantly for shorter tweets (-1 SD; β=.17,p<.001). The finding that the GDD predicts coders' ratings of greed for tweets one standard deviation below the mean in length gives confidence that this tool is valid for tweets of any length, because tweets one-standard deviation below the mean were only 8.25 words on average.

Convergent Validity

To assess convergent validity of the GDD, we pre-determined a list of dictionaries from LIWC-22 that capture related constructs based on the extant literature on greed. We pre-registered this list prior to conducting analyses (https://bit.ly/3rWOw3N). We pre-registered tentative predictions, informed by research team consensus and intuitions, in the absence of any existing empirical (or – for that matter – theoretical) research that could have informed these hypotheses. We tested for associations between constructs that are theoretically positively associated with self-reported greed, but we believe these predicted associations should be viewed as somewhat exploratory due to the dearth of knowledge about the correspondence between associations between self-report measures and linguistic markers of those same constructs (8).

We did not make confirmatory hypotheses about constructs negatively associated with greed because it is difficult to predict whether constructs that are negatively associated when assessed with self-report measures (e.g., greed and altruism) would show those same associations in text (8). It is possible that text mentions of constructs that are experienced in opposition would be positively correlated (e.g., a text source that describes greed might discuss how greedy people are not altruistic, resulting in a positive correlation between linguistic markers of greed and altruism).

Nonetheless, we pre-registered the prediction that positive associations would emerge between the GDD and the LIWC-22 dictionaries for the following constructs (see [7] for brief descriptions of each LIWC dictionary):

- Achievement
- Acquire
- All-or-none
- Clout
- Discrepancy
- Money
- Need

- Power
- Quantities
- Reward
- Risk
- Want
- Work

We tested the association between the GDD and these dictionaries in the sample of Senators' tweets (N = 861,104) described in the main text. Of these 13 dictionaries, 10 showed the expected positive relation with the GDD ($\beta_{Acquire}$ =.19, $\beta_{Discrepancy}$ =.08, β_{Money} =.19, β_{Need} =.13, β_{Power} =.03, $\beta_{Quantities}$=.31, β_{Reward} =.05, β_{Risk} =.02, β_{Want} =.14, β_{Work} =.15; all *ps*<.001), and the remaining three showed unexpected negative relations ($\beta_{Achievement}$ =-.02, $\beta_{All\text{-}or\text{-}none}$ =-.02, β_{Clout} =-.03, all *ps* < .001). We also tested whether these associations held after removing shared words between the GDD and the other dictionaries. All of the significant associations remained significant and in the same direction, except for Power and Want, which became negatively associated with the GDD after removing shared words (β_{Power} =-.03, β_{Want} =-.01; all *ps* < .001). To obtain additional validation data, we tested these same associations in an open dataset of 2,225 BBC articles (https://www.kaggle.com/c/learn-ai-bbc). The associations were generally in the same directions, although some were non-significant due to the smaller sample size. More specifically, all of the 10 dictionaries that showed positive relations with the GDD in the Twitter data showed positive relations here, except for Power: $\beta_{Acquire}$ =.19, p < .001; $\beta_{Discrepancy}$ =.04, p = .06; β_{Money} =.40, p < .001; β_{Need} =.003, p = .87; $\beta_{Quantities}$=.37, p < .001; β_{Reward} =.03, p = .11; β_{Risk} =.02, p = .34; β_{Want} =.11, p < .001; β_{Work} =.22 p < .001. The three dictionaries that showed negative correlations in the Twitter dataset also showed negative relations here, along with Power: $\beta_{Achievement}$ =-.11, $\beta_{All\text{-}or\text{-}none}$ =-.09, β_{Clout} =-.09, β_{Power} =-.07; all *ps* < .001. After removing shared words, Discrepancy, Reward, and Want showed negative relations with the GDD ($\beta_{Discrepancy}$=-.06, p = .003; β_{Reward} =-.07, p < .001; β_{Want} =-.10, p < .001).

Taken together, these results support the validity of the GDD and suggest that it adequately captures and discerns mentions of greed in diverse sources of contemporary text. More work is necessary to examine the generalizability – and potential boundary conditions – of the GDD's validity for assessing greed in other text forms, including texts from other historical periods.

References

1. M. A. Lawson, A. E. Martin, I. Huda, & S. C. Matz, Hiring women into senior leadership positions is associated with a reduction in gender stereotypes in organizational language. *Proceedings of the National Academy of Sciences*, **119**, e2026443119 (2022).
2. T. G., Seuntjens, M. Zeelenberg, S. M. Breugelmans, & N. van de Ven, Defining greed. *British Journal of Psychology*, **106**, 505–525 (2015).
3. E. Rosch, Cognitive representations of semantic categories. *Journal of Experimental Psychology: General*, **104**, 192-233 (1975).
4. B. Fehr, Prototype analysis of the concepts of love and commitment. *Journal of Personality and Social Psychology*, **55**, 557-579 (1988).
5. B. Fehr, & J. A. Russell, Concept of emotion viewed from a prototype perspective. *Journal of Experimental Psychology: General*, **113**, 464-486 (1984).
6. S. X. Luo, F. Van Horen, K. Millet, & M. Zeelenberg, What we talk about when we talk about hope: A prototype analysis. *Emotion*, **22**, 751 - 768 (2022).
7. R. Boyd, A. Ashokkumar, S. Seraj, & J. Pennebaker, *The Development and Psychometric Properties of LIWC-22*. https://doi.org/10.13140/RG.2.2.23890.43205 (2022).
8. V. Ponizovskiy, M. Ardag, L. Grigoryan, R. Boyd, H. Dobewall, & P. Holtz, Development and validation of the personal values dictionary: A theory–driven tool for investigating references to basic human values in text. *European Journal of Personality*, **34**, 885–902 (2020).
9. F. Faul, E. Erdfelder, A. Buchner, & A.G. Lang, Statistical power analyses using G*Power 3.1: Tests for correlation and regression analyses. *Behavior Research Methods,* **41**, 1149–1160 (2009).
